# The challenges of integrating signposting into general practice: qualitative stakeholder perspectives on care navigation and social prescribing in primary care

**DOI:** 10.1186/s12875-022-01669-z

**Published:** 2022-04-01

**Authors:** Lisa Brunton, Abigail Tazzyman, Jane Ferguson, Damian Hodgson, Pauline A. Nelson

**Affiliations:** 1 School of Health Sciences, Faculty of Biology Medicine and Health, University of Manchester, Oxford Road, M13 9PL Manchester, UK; 2 Sheffield Methods Institute, Interdisciplinary Centre of the Social Sciences, University of Sheffield, 219 Portobello, S1 4DP Sheffield, UK; 3 Alliance Manchester Business School, University of Manchester, Booth St West, M15 6PB Manchester, UK; 4 Sheffield University Management School, Conduit Road, S10 1FL Sheffield, UK

**Keywords:** General practice, Signposting, Care navigation, Social prescribing link worker, Qualitative research

## Abstract

**Background:**

A national policy focus in England to address general practice workforce issues has led to a commitment to employ significant numbers of non-general practitioner (GP) roles to redistribute workload. This paper focuses on two such roles: the care navigation (CN) and social prescribing link worker (SPLW) roles, which both aim to introduce ‘active signposting’ into primary care, to direct patients to the right professional/services at the right time and free up GP time. There is a lack of research exploring staff views of how these roles are being planned and operationalised into general practice and how signposting is being integrated into primary care.

**Methods:**

The design uses in-depth qualitative methods to explore a wide range of stakeholder staff views. We generated a purposive sample of 34 respondents who took part in 17 semi-structured interviews and one focus group (service leads, role holders and host general practice staff). We analysed data using a Template Analysis approach.

**Results:**

Three key themes highlight the challenges of operationalising signposting into general practice: 1) role perception – signposting was made challenging by the way both roles were perceived by others (e.g. among the public, patients and general practice staff) and highlighted inherent tensions in the expressed aims of the policy of active signposting; 2) role preparedness – a lack of training meant that some receptionist staff felt unprepared to take on the CN role as expected and raised patient safety issues; for SPLW staff, training affected the consistency of service offer across an area; 3) integration and co-ordination of roles – a lack of planning and co-ordination across components of the health and care system challenged the success of integrating signposting into general practice.

**Conclusions:**

This study provides new insights from staff stakeholder perspectives into the challenges of integrating signposting into general practice, and highlights key factors affecting the success of signposting in practice. Clarity of role purpose and remit (including resolving tensions inherent the dual aims of ‘active signposting’), appropriate training and skill development for role holders and adequate communication and engagement between stakeholders/partnership working across services, are required to enable successful integration of signposting into general practice.

**Supplementary Information:**

The online version contains supplementary material available at 10.1186/s12875-022-01669-z.

## Background

Declining numbers of general practitioners (GPs) in England, together with an ageing population living with increasingly complex health needs, fuels the challenge of providing sufficient capacity to meet the demand for primary care [1]. This has led to a national policy focus on addressing workforce issues in this setting. Alongside efforts to increase the number of GPs, a key priority in recent years has been the integration of non-GP roles into general practice to redistribute GP workload [2]. For many years, practice nurses have been working alongside GPs, and more recently a range of other health professionals, such as nurse practitioners, pharmacists and physician associates have been employed with the aim of taking on work previously conducted by GPs. More recently, the National Health Service Long Term Plan (NHS LTP) [3] and the General Practice Contract Five Year Framework [4] set out the intention to create an additional 20,000 full-time equivalent non-GP roles in general practice over the next five years, funded via the Additional Roles Reimbursement Scheme (ARRS) [5].

In this paper, we focus on two non-GP roles that have featured in recent policy arrangements [2, 3]: the care navigator (CN) and social prescribing link worker (SPLW) roles, which both aim to introduce the concept of ‘active signposting’ (or ‘signposting’) into the general practice setting. Active signposting is one of the ‘10 High Impact Actions’ to release capacity in general practice (i.e. to reduce the workload of GPs), and aims, at the first point of contact, to signpost patients to the ‘right’ professional or service at the ‘right’ time [2].

CNs originated in the United States of America (USA) as a role to support socially disadvantaged groups to access health services, and a range of different professionals (e.g. nurses and social workers) now work as CNs in the USA system [6]. In the United Kingdom (UK), by contrast, the CN role is usually undertaken by practice receptionist staff or practice managers as an enhancement to their existing roles, with’active signposting’ as the foundation of the role. According to NHS England guidance, the CN role aims to achieve several goals: to free up 5% of GP consultations; make the most appropriate use of the non-GP workforce; increase receptionists’ job satisfaction; and make it easier for patients who require a GP appointment to access one [7]. From 2017, £45 million was made available over five years to Clinical Commissioning Groups (CCGs) to train receptionists and clerical staff to undertake enhanced roles in active signposting, by becoming CNs, and managing clinical correspondence [7]. Whether the CN role is meeting these expressed aims is currently unclear. One small-scale peer reviewed study [8] and two case studies [9, 10] suggest that care navigation can reduce the number of ‘potentially avoidable’ [8] or ‘inappropriate’ [9] GP appointments carried out by practices. For example, over a 7-month period, care navigation was calculated to have saved 1,685 GP appointments across an area in the North of England [10]. Other evidence suggests that the signposting of patients who are judged not to require a GP appointment to allied health professionals, non-medical staff or alternative services has the potential to improve patients’ access to care and enable their problems to be resolved more quickly [8]. However, more robust outcomes evidence is needed to see how the role is impacting on the wider primary care system. Additionally, literature on the process of how CN roles are functioning in general practice, from the perspective of staff, is absent.

The SPLW role is one supported by the ARRS [5], with the NHS LTP [3] committing to employing 1000 new SPLWs by 2020/21. The aspiration is that approximately one million patients will have accessed social prescribing in some form by 2024 [11]. Funding will be directed through Primary Care Networks (PCNs) made up of groups of general practices in the same geographical area, that will provide care to populations of between 30,000 and 50,000 patients each [5]. Notably, there is no single definition of social prescribing and different models have been adopted across different areas [12]. While distinctions are made between SPLWs and receptionists undertaking CN (for example, see the CN competency framework [13] which outlines three tiers of navigation: essential, enhanced and expert, and the type roles and competencies required at each level), some models do use the terms CN and SPLW interchangeably [6]. In addition, SPLWs often have different titles (such as ‘care navigator’, ‘community navigator’ or ‘community connector’) in different settings/locations [14]. Indeed, one study identified 75 different titles for this type of role [15]. However, all SPLW roles have an element of signposting at their base [16, 17] as the aim is to link or connect patients with community-based resources [18] (such as welfare advice, bereavement support, health behaviour change programmes, or creative activities) [14]. Similarly to the CN role, the SPLW role broadly involves the referral of patients with social, emotional or practical needs to non-clinical services, but goes further, in aiming to provide holistic, community-based support through shared decision-making (based on what matters to the person), personalised care & support planning [14, 19]. SPLWs, in common with CNs, are also conceptualised as a way of reducing the number of GP consultations, while simultaneously improving patients’ physical and/or mental health [14]. The evidence to support the effectiveness of social prescribing in relation to these outcomes is equivocal however. There is some evidence to show that social prescribing can lead to a range of positive health and well-being outcomes; for example, increased quality of life and subjective health [20], and feelings of increased control and self-confidence, reduced self-isolation and reported positive health-related behaviour changes such as increased physical activity, weight loss and healthier eating [21]. However, a widely-cited systematic review published in 2017 [22], found a lack of robust, quality evidence that social prescribing reduces primary care consultations or improves patients’ physical and/or mental health. The review also highlighted that included studies were mainly small scale, poorly designed evaluations. This systematic review was recently updated by Public Health England and it reached the same conclusions [23]. Additionally, a number of studies have examined how social prescribing services are operating. These focus on a range of process issues such as the enablers of and barriers to implementation, delivery, uptake and sustainability of social prescribing services [14, 17, 18, 24, 25].

Despite both the enthusiasm and policy drive to integrate the CN and SWLP roles into general practice, there is an absence of research exploring how these specific roles are being planned and operationalised in this setting, by including the perspectives of role holders and other staff stakeholders. In particular, the notion of how active signposting within these roles is being integrated into general practice has not been examined, and this is the focus of our study.

## Methods

The data we report in this paper form part of a wider study that explored the integration of new non-GP roles into general practice in Greater Manchester, a region in England, investigating how roles were being established and implemented [26]. This paper focuses on the challenges of integrating ‘signposting’ into general practices, from the perspectives of stakeholder staff involved in the roles of CN and SPLW. An earlier version of this paper was submitted and presented at the Health Services Research UK 2020 conference [27].

This study used in-depth, qualitative methods to understand participant perspectives on the CN and SPLW roles. Ethical approval was gained from a University of Manchester Research Ethics’ Committee (reference number 2017–2619-4613). An interview topic guide (see Supplementary file 1) was developed from a previous literature review [6] and questions focused on participants’ perceptions of the aims and objectives of the roles, how roles had been introduced to general practice, the challenges and opportunities in implementing the roles and their views on the sustainability of roles and services. We used purposive sampling to gain a wide sample of stakeholder staff involved in CN and SPLW roles including those in service lead roles, role holders (e.g. receptionists undertaking care navigation; SPLWs) and host general practice staff (such as GPs and practice managers). Data were collected between April and July 2019 by LB and AT in semi-structured interviews and one focus group; data collection was conducted face-to-face, except for a small number of interviews via telephone at the request of respondents. Interviews were audio-recorded (with permission), transcribed, anonymised and uploaded to the NVivo 11 qualitative data software programme [28]. Data analysis and data collection were conducted concurrently and the data was analysed using a Template Analysis approach [29]. Data was first coded to a template developed from conducting previous research on new roles. Examples of categories included impressions of new roles and their fit with general practice and the barriers and enablers to implementation. Codes were then critically scrutinised to develop broader categories. Throughout, we used the constant comparative method [30] to identify similarities and differences within and across the data to expand the boundaries of categories. Categories were discussed and refined through regular meetings with the research team (LB, AT, JF, DH and PAN) in order to generate the interpretive themes outlined below.

## Results

### Participants

A total of 34 respondents took part in 17 semi-structured interviews and one focus group. The focus group comprised 14 practice managers with experience of the CN role. In two of the interviews, more than one person was interviewed at the same time. See Table 1 for sample characteristics.

**Table 1:** Sample characteristics*

Participant role	Number of participants
CN training/service lead (including 1 GP)	2
CN role holder	5
CN host practice staff (1 assistant practice manager, 15 practice managers, 1 GP)	17 (*n* = 14 practice managers in focus group)
SPLW service lead	5
SPLW role holder	4
SPLW host practice staff (GP)	1
**Total**	**34**

^*^ *CN* data from one area of Greater Manchester, *SPLW* data from five areas of Greater Manchester

### Key themes

Three key themes are presented, highlighting the challenges of integrating signposting into general practice, from the perspectives of respondents involved in CN and SPLW roles: 1) role perception; 2) role preparedness and; 3) integration and co-ordination of roles.

The three themes highlight challenges which are common to both roles, but which play out differently within each role.

#### 1. Role perception

Firstly, signposting was made challenging by the way both roles were perceived by others (e.g. the public, patients and general practice staff). Concerns were raised by respondents that stakeholders were often not well enough informed about the CN and SPLW roles, with implications for the success of roles, including the willingness of general practices to take them on:

Practice manager 3: *There are all these new services coming into the community or new procedures, like the care navigation […]. The problem is educating the patients…*

Practice manager 2: *there’s nobody telling the patients the face of your NHS does have to change because we can’t cope with health at the moment.* CN focus group, area 3.

SPLW host 1 (GP): *Some [colleagues] just were very sceptical. I know a GP colleague in my own health centre would say, “Are you going off singing? Some of us are practising proper medicine”... So, I think, there is some scepticism… there is some cynicism [about setting up social prescribing roles in general practice].* SPLW interview*,* area 10.

The CN role was explained by respondents as a way to enable patients to be signposted at the first point of contact, to the ‘right’ professional or service. However, respondents simultaneously acknowledged that a key driver underpinning the CN role was to free up GPs’ time by directing patients to other sources of help:

CN host 15 (assistant practice manager): *I see [care navigation] as being able to direct patients to the most appropriate service. I think a lot of the calls that we get through don’t need to actually be seen by a GP. But I think the default stance as a patient [is] to ring your doctor’s surgery when you’re not feeling very well.* CN interview, area 3.

Further, respondents reported that the CN role was perceived by some patients as merely a way to signpost them away from GP appointments, rather than steering them towards the most appropriate care. Respondents described how their attempts at signposting patients away from GPs could lead to challenging and uncomfortable interactions:

Receptionist 1 (CN): *The majority of [patients] don’t like it, because they want to hear that coming from the doctor. If they feel they need to go somewhere else, they want the doctor to tell them that, not the care navigator.* CN interview, area 3.

Receptionist 4 (CN): *So we have had quite a bit of abuse, but now like I say [patients] are getting used to it, but they were like, “why should I tell you, you’re not medically trained”, and you get that from them.* CN interview, area 3.

Additionally, some respondents reported a lack of engagement by GPs in relation to integrating the CN role into their practice, suggesting that greater and more prolonged engagement work over time was needed to highlight the perceived value of signposting and to support implementation of the role:

CN training/service lead 1 (GP): *Certainly the biggest frustration…was the lack of GP support, and them not valuing it…if you haven’t got the people at the top of the chain engaged, it’s not really going to work. So yeah, we still need to work on that, and we keep chiselling away…but it’s like an oil tanker, you wouldn’t believe that this was actually part of the 10 Point Plan to help you.* CN interview, area 3.

The perspectives of respondents involved in the SPLW role indicated a greater emphasis on it being a way to improve patient care and to address wider social and psychological needs of patients; there was a strong focus on the role being perceived as person-centred and holistic in nature. While signposting people to community assets was considered to be a core part of the SPLW role, it was emphasised that appropriate signposting could only be done by first establishing trusting relationships with clients to enable adequate needs assessment, and where relevant, use motivational interviewing and/or behavioural change techniques to enable change:

SPLW role holder 1: *…because a lot of people are struggling to engage with the world around them, struggling to leave the GP surgery and go elsewhere. So that assessment bit and that alliance that you build with someone is crucial to understanding what the underlying issue is… we’re almost like social prescribing but we also would provide intervention…around behavioural change as well. Because the idea being that these might be repeat presenters to their GP. They’re not just going to go somewhere because you tell them…*

SPLW service lead 1: *Yes, mostly you’re intervening to… as the kind of a connection, rapport building and assessment element of it. And so that’s a good bolt onto the whole social prescribing service for us, I think.* SPLW interview with two respondents, area 10.

Yet, respondents described how a lack of role clarity amongst general practice staff presented challenges to signposting in a different way, because patients were often inappropriately signposted to SPLWs. While SPLWs did accept referrals for people with mental health issues, *“mental health is the biggest reason for referral into the service. That is mainly mild to moderate depression and anxiety”* (SPLW service lead 4, area 1), a number of respondents described how they received referrals that their social prescribing service was not equipped to manage, such as people in mental health crisis:

SPLW service lead 5: *… [person had] literally tried to commit suicide the day before, the day after we had a referral through, to say, can we provide this lady with some support? To which we’d have to say, we just politely reminded [the referring staff] that we’re not [mental health] professionals, we’re not counsellors…it’s important [the person] gets that treatment first, before we look at [social prescribing].* SPLW interview, area 7.

A lack of understanding of CN and SPLW roles among the public, patients and general practice staff challenged the successful integration of signposting into primary care.

#### 2. Role preparedness

Signposting was made demanding by a second challenge, related to complications around a lack of training for both roles.

Some receptionists considered the upskilling of their role to CN as an opportunity for career development and welcomed the chance to take on this enhancement; although taking on the enhanced role was not remunerated with an increase in salary. However, others perceived the CN role to have been foisted upon them and were less enthusiastic about undertaking signposting. For example, one respondent described how the decision to bring in the CN role was not a decision made by their practice:

Receptionist 5 (CN): *From memory, I think it’s about two years ago and I understand, I think it was the CCG [clinical commissioning group] that brought it in… it certainly wasn’t our decision to do it.* CN interview, area 3.

GPs within the sample suggested that care navigation was a form of non-clinical triage and considered it a safe way to direct patients to the correct clinician:

Interviewer: *And does the care navigator role cross over with any other roles do you think, any that kind of exist within primary care, or is it quite distinctive?*

CN training/service Lead 1 (GP): *I think it’s…I mean, because it’s non-clinical triaging I suppose if you wanted to say it that way, but I think historically in primary care everything has been…the GP or the nurse or whoever who’s done triaging [of] any description historically. So I think it’s giving them [receptionists undertaking care navigation] a label but giving them a safe label.* CN interview, area 3.

However, receptionists’ reluctance to embrace the CN role was said to be due to feeling unprepared to carry out signposting, not only because of fear that it could lead to conflict with patients, but also because some perceived active signposting as a form of clinical triage. This led to concerns regarding patient safety. Some receptionists perceived that the CN role was not suitable to be carried out by non-clinicians:

Receptionist 5 (CN): *I think, we all still feel a little bit that we may be making the wrong decisions for the patient because we aren’t clinical. I feel it should be…at some point, there should be somebody clinical doing this triage and signposting, because it’s easy for somebody to miss [a serious health issue] without clinical knowledge.* CN interview, area 3.

Prior to taking on the CN role, receptionists within area 3 had often experienced delays in access to care navigation training, or received no formal training at all in how to signpost patients. This could exacerbate feelings of unpreparedness for the role. Some respondents who had been working as care navigators before undertaking training described how the course had increased their confidence in knowing how to handle patients who were resistant to being signposted away from GP appointments, and indicated that they were tasked with passing training tips on to their practice colleagues:

Receptionist 4 (CN): *Well, whenever we started the care navigating, me and my colleague didn’t go on the course till a long time after, like months after. So we had nothing beforehand and that was only two of us out of the whole reception team, so the rest of them haven’t had that one day training course like we have, so…[…] we got some tips back from [training course] and they seem to have worked better now that we changed what we were saying on the phones to the patients. But yeah, we had to come back and tell all the group, so yeah, I think everyone should have really had the training though.* CN interview, area 3.

The SPLW role was a relatively new role being undertaken across the Greater Manchester region. Role holders often came from a variety of different professional backgrounds (for example, housing, debt management, exercise or health coaching), meaning that they often brought different training and skills to the SPLW role:

SPLW role holder 3: *Because obviously, there’s a number of workers and we’re all from… different backgrounds and different areas of what we’ve worked in previously. Which is good. So, I’ve come from drug and alcohol and criminal justice, we’ve got somebody who’s come from diabetes, we’ve got somebody who’s come from dementia. So, if we have any issues, we can contact them...* SPLW interview, area 10.

While this variation in skillset and expertise within teams was highlighted as a positive aspect, it also raised concerns that it could affect the consistency of offer across areas, meaning that signposting and other aspects of the role were undertaken differently by different professionals:

SPLW service lead 1: *…we’ve been very focused… on equity of provision…that to us is very difficult to meet… So [one SPLW] might lean more on mental health, [another] might lean more into supporting with debt… and from my point of view as a service lead that’s very, very difficult to manage because I can’t actually say that we’re delivering exactly the same thing in each neighbourhood. Whereas if they asked the physio team, they’re all delivering physio or the pharmacy team are all delivering medication reviews.* SPLW interview with two respondents, area 10.

To address the variation in service offer, some service leads were developing competency frameworks and/or providing training to ensure a minimum skillset across all SPLWs in an attempt to standardise the service offer across their patch:

SPLW service lead 5: …*part of the CPD [continuing professional development] and support that we provide our members of staff, is that they go and shadow others, working from different areas. Like I say, we drag in professionals that can give us some sort of CPD, so mental health awareness, suicide awareness, all those types of activities…so there’s an extensive training list that our members of staff have been on…to help them cope with various issues and pressures.* SPLW interview, area 7.

The successful integration of signposting into general practice was made challenging by respondents feeling unprepared to take on the roles of CN and SPLW. Meeting training needs to ensure role holders were confident and competent to undertake their roles was deemed important to the success of signposting.

#### 3. Integration and co-ordination of roles

A third signposting challenge relates to the integration and co-ordination of roles, and suggests that the success of CN and SPLW roles is reliant on the inter-relationship between wider services and systems. A lack of planning and co-ordination across services could challenge the success of signposting.

A disconnect between primary care and other parts of the health and social care system could have a negative effect on the signposting activity of CNs. There were reported instances of CNs signposting patients to already over-loaded services that did not have the capacity to accept them, leading to lack of patient trust in the competence of CN receptionists (and by association threatening patient trust in general practice). Notably, this also created dissatisfaction for role holders themselves, as highlighted in the following dialogue:

Practice manager 4: *Talking about signposting, I believe services to which you’re signposting need to be very robust and ready for it.*

Practice manager 7: *We found that some [community services] couldn’t deal with the impact.* […]

Practice manager 1: *But that frustrates the patients then.*

Practice manager 2: *I was just going to say [that].*

Practice manager 4: *…they’re batting back to our service.*

Practice manager 2: *So, the patient loses faith in the call handler because they think that [they’ve] just been fobbed off. “You can’t even get through to that doctor’s, and then when you do she’s telling me that I need to go there, and then I’m phoning there again and then to be told that they’re fully booked up”. And then it gives us a bit of a bad…it’s not fair really. CN focus group, area 3.*

Similarly, a lack of co-ordination and integration of the primary care-based SPLW role with other roles and services in the system was reported to impact on the success of signposting. Social prescribing schemes could sometimes overlap with other wellbeing services causing confusion for general practice staff about where to refer patients and could cause professional tensions with other services:

SPLW service lead 1: *When we began, there’s another service that’s quite similar to ours and again there was some political but…they felt like our service was a threat to their service. And yeah, that was a challenge. I think we’ve got there now, but…* SPLW interview with two respondents, area 10.

Respondents involved in SPLW roles asserted that successful signposting and social prescribing were predicated on the strong relationships between three inter-connected parts of the system, each with its own purpose: primary care referring patients to SPLWs; SPLWs supporting patients; and the voluntary, community and social enterprise (VCSE) sector providing services. A focus on one single element without reference to the other parts could affect the success of channelling patients to the right help:

SPLW service lead 2: *How does the wider VCSE link into social prescribing? What are the relationships like? How resilient is it? How ready are groups to take referrals? How strong and resilient are they? How much funding goes into those groups? …all of that is social prescribing. We generally look at the link worker alone, primary care alone. The whole thing, without any of those three things it doesn’t work.* SPLW interview, area 4.

Inter-organisational working was particularly enhanced in one area. The SPLW service was embedded within both the community and local hospital multi-disciplinary teams, and SPLWs attended regular meetings for clients who had more complex needs so that they could be referred to relevant services more quickly, to prevent crisis and avoid duplication of care. This service was one of the longest established SPLW services within the Greater Manchester region.

SPLW role holder 2: *If there's anything that we receive and it's quite complex, and there's not one specific service to link up with, that's when we go to the [multidisciplinary meetings]. Because at the …meetings which are once a week, which we all attend, there's all services there, so there's social care, there's mental health, housing, DWP [UK Department for Work and Pensions]. So we can bring a case up… and then you've got input from all services. And it just avoids duplication. Because I might be saying to someone, we'll get you a social worker or we'll get somebody else involved, and then when you take it to this meeting, they've got all that.*

*[…]*

SPLW service lead 4*: And also, if there's mental health involved, someone who's not normally at the meeting, then they would get those people at the meeting, so that there can be a proper discussion and problem solve, and get a proper plan going. So that works really well because the clinical person or social care [worker] will be given the case-holder role and then [SPLW] or recovery services [worker] will be given the key worker role. And they very much liaise and then feedback at the next meeting. It works really well.* SPLW interview with three respondents, area 1.

In another area, a social prescribing scheme seconded members of staff from the VCSE sector to work as SPLWs between organisations. This led to a more comprehensive understanding of what services were available and how patients could be supported to access them:

SPLW Service Lead 5: *We have a really good understanding of what’s available in the community voluntary sector… Their expertise and knowledge, [it’s] the best part of 20/30 years that those companies have been in established in [area name], in the community voluntary sector.* SPLW interview, area 7.

However, with the introduction of the primary care-based SPLW roles funded through PCNs, service leads raised concerns (in particular, from those operating social prescribing services within the VCSE sector) about how primary care based SPLW roles would operate alongside the more long-standing SPLW roles already in the community. Respondents were apprehensive that the SPLW role, including the signposting aspect, could become narrowly clinically focused, less holistic and more focused on reducing GP workload rather than being patient-centred as the role was originally intended.

SPLW service lead 3: *They could end up having a social prescriber in each GP practice, being part of the clinical team with the consequences of that… if you work within a clinical team, you are going to become more clinical… I’d like to see that [social prescribing] stays with community organisations, to keep the roots where the roots need to be.* SPLW interview, area 4.

Respondents also questioned whether existing community-based social prescribing schemes would continue to be funded, in light of the introduction of PCN-funded SPLW roles. This led to questions over how future social prescribing models would sustain and secure funding for the VCSE sector in order to provide the services to which patients could be signposted. In addition, there was concern that the intelligence and community links built over time by existing schemes could be lost, as in some areas there was evidence that SPLWs funded through PCNs were being employed without any input from existing schemes.

SPLW service lead 5: *… how are we going to pass all this information on, if more funding doesn’t come in? How can the PCNs learn from what we’ve learned? … because it’s valuable, and what sort of learning…and carry on the good work that we’ve done, and let’s see how we can support you, and ultimately sustain social prescribing in [our area].* SPLW interview, area 7.

The integration of signposting into general practice was challenged in a third way by a lack of co-ordination across services and systems. For the CN role, this led to patients being directed to services that did not have the capacity to match demand and patients were bounced back to general practice. For SPLW role, while one area’s well-established SPLW service reported being successfully integrated with multi-disciplinary services, most areas were struggling with a lack of planning and co-ordination with existing services. This led to a lack of knowledge and information sharing across services and an overlap of service delivery within areas.

## Discussion

### Summary of findings and comparison with existing literature

This study is the first to examine staff perspectives on how signposting is planned and operationalised in English general practice in the context of two new non-medical roles – CNs and SPLWs – both of which involve signposting as a key task.

This work provides new insights into the issues raised when attempting to integrate signposting in this setting, and highlights key factors—role perception, role preparedness and integration/co-ordination of roles and services—that affect how well signposting can be operationalised into practice. As such, our work responds to prior calls in the literature for a closer examination of the role of receptionists undertaking ‘active signposting’ in general practice [31] and of process issues associated with social prescribing [17].

#### The importance of clarity of purpose in CN/SPLW roles

Our work firstly highlights that a lack of clarity about the purpose of these roles may impinge upon the success of signposting by generating suspicion among patients and general practice staff about the ability and suitability of CNs and SPLWs to undertake work in general practice. Role ambiguity also appeared to affect the willingness of GPs (who, in the UK, are not only clinicians but business owners/employers) to take on such roles, a finding which supports prior research on other skill-mix changes that have been introduced in recent times into general practice [32]. A recent realist review [31] aimed to understand how ‘connector schemes’ (delivered by CNs or SPLWs) worked *‘for whom, why, and in what circumstances?’* (p.1). In parity with our findings, the review highlighted the importance of ‘buy-in’ from all stakeholders, including patients, commissioners and general practice staff, in order that CN and SPLW services can be ‘legitimised’ and accepted into primary care. The authors identified that clear information about the role and remit of SPLWs was necessary to avoid confusion and inappropriate referrals. Our study offers empirical evidence to support this concern, highlighting that confusion about role remit did indeed arise, leading to patients in need of more specialist management being inappropriately referred by practice staff to SPLWs (in effect, generating a form of inappropriate ‘reverse’ signposting). This lack of clarity also manifests in the inherent tension uncovered by our study between the dual aims in both roles of 1) driving down the number of GP appointments filled and 2) signposting patients to the ‘right’ professional at the ‘right’ time. We found that diverting patients rapidly, at the first point of contact, away from GPs to other services could result in tense exchanges with patients, and create disharmony, with implications for the success of signposting. Signposting patients away from GP appointments was said to require trust to be built up over time between role-holders and patients and involved skilled assessment of patients’ needs. Previous research has also indicated that rapport and trust with service users is a prerequisite to accurately identifying their needs and linking them successfully into other services [21]. Other authors have also suggested that even if patients initially comply with attempts to divert them away from GPs, if they are dissatisfied, this may lead to further efforts to see a GP and a ‘revolving door’ of consultation requests, potentially increasing rather than reducing staff workload [15].

#### The need for adequate training and skills development for role-holders

Secondly, we demonstrate how signposting can be challenged by a lack of training and skills development for CNs and SPLWs. Where extended care navigation duties had been imposed upon staff (and notably without a corresponding increase in remuneration), rather than undertaken by choice, this affected role holders’ sense of job satisfaction. Even when staff were willing to extend their roles, they could feel ill-equipped to adequately assess patients’ needs and appropriately channel them to sources of help. These feelings were exacerbated by the fact that some staff had received no formal training before taking on the role of CN, highlighting an inconsistency in CN training provision, at least, across one area within Greater Manchester. This is despite national guidance [13] setting out the knowledge, skills and behaviours required for an individual to be able to perform care navigation and for organisations to plan care navigation training.

Other studies have highlighted the importance of training and skills in CN and SPLW roles. The success of a CN intervention evaluated across two practices [8] was influenced by staff training in the use of the CN protocols; a realist review [17] identified that sufficiently trained and knowledgeable link workers enabled the successful transition of patients between services.

The importance of staff receiving sufficient support and training is highlighted in a recent survey conducted by the National Association of Link Workers to understand the knowledge, skills, experiences and support needs of link workers in the UK [33]. A total of 279 social link workers completed the survey, of which 221 were based in England. The survey identified that almost one third of responders answered ‘yes’ to the question: *‘have you or might you consider resigning in the next year or so due to a lack of supervision or support?*’, 61% reported receiving no clinical supervision, while 11% received no support in any form. The survey could be considered a prescient warning, given the vital role that SPLWs have reportedly played in primary care’s efforts towards supporting vulnerable patients during the Covid-19 pandemic [34, 35]. It has been suggested that the Covid-19 pandemic has exacerbated issues of lone working for link workers who work with people with complex needs [33], and highlights, alongside our own study, the importance of ensuring sufficient supervision and training to help those in such roles feel supported.

While the lack of adequate training in signposting for CNs has been highlighted previously, more troublingly, our study shows that staff in these roles often felt that assessing patients’ needs was a clinical task, akin to triage and one that was said to be better carried out by trained clinicians. CN staff had grave concerns about failing to recognise serious health issues when patients presented for help, and risking patient safety by inappropriately diverting them away from GP appointments. Although it has been highlighted previously that any signposting should privilege patient preference and safety, it is also evident that even experienced clinicians vary in their assessment of what constitutes a potentially avoidable appointment request [8]. This raises concerns about the advisability of CN receptionists managing risk in the unpredictable setting of general practice, where even qualified and experienced clinicians must develop risk-management skills over time [31]. For SPLW role-holders, variations in training led to a lack of consistency in the type of signposting/social prescribing that could be offered across an area, with fragmented, local attempts at standardising competency frameworks and training, in an effort to streamline the service offer. Recent national guidance may go some way to alleviate this issue; for example, the ‘PCN reference guide for social prescribing’ sets out PCNs’ responsibilities to ensure SPLWs undertake specific training requirements to standardise learning and development [36].

#### Planning for and coordination of effects across the wider system

Thirdly, this work highlights that a disconnect between different parts of the health and social care system has implications for successful signposting. Patient trust in the competence of CN role-holders to assess their needs and channel them appropriately was threatened when patients were re-directed to over-subscribed services with little or no capacity. This was stressful for role-holders and increased feelings of job dissatisfaction. The possibility of such unintended consequences arising has been raised previously [37]. A lack of integration between parts of the system (specifically, primary care, social prescribing services and the voluntary sector) also affected the success of signposting for SPLW role-holders, when primary care-based social prescribing services overlapped with other wellbeing services, causing tension. The importance of actively managing the introduction of new roles into primary care to avoid this kind of duplication and/or inefficiency caused by ‘transaction’ costs has previously been recognised [38]. Collaborative relationships between different sectors, good service infrastructure and communication with clear referral processes have been highlighted previously as key ingredients for the successful implementation of social prescribing services [25, 39]. Indeed, where reports of inter-organisational working in this study were strong, signposting/social prescribing was also said to work better. Finally, there were also concerns, particularly among already existing voluntary sector SPLWs, that the new primary care-based SPLW role would change the fundamental nature of social prescribing, moving it away from the role’s traditionally ‘holistic’ roots towards a more clinically-focused role which focused on helping GPs to reduce their workloads rather than privileging the wider psychological and social needs of patients. Our previous work on new roles signals the need for consideration/anticipation of the potential wider system and governance effects of making these skill-mix changes [38]. Further, the findings of the present study chime with Fixsen and colleagues’ insight that balanced management is needed in the multi-stakeholder arena of social prescribing, to prevent the interests of one group of stakeholders taking precedence over others’ [24].

### Strengths and limitations

This is the first study to explore in depth the challenges of integrating signposting into general practice from the perspectives of a wide variety of stakeholder staff involved in CN and SPLW roles (i.e. services leads, receptionists undertaking CN duties, SPLWs and general practice host staff), which have previously been missing from the literature. The qualitative perspectives gathered illuminate the planning and operational issues that arise when integrating signposting into general practice.

We used a mixture of interview and focus group data collection methods to gain staff perspectives. In two instances, respondents were interviewed together (we conducted one interview with two respondents – an SPLW role holder and their SPLW service lead; and one interview with three respondents – an SPLW administrator, an SPLW role holder and their SPLW service lead). While the power differential between respondents could have potentially inhibited the responses of those in more junior roles, the staff in these interviews appeared willing to comment openly on the challenges they had experienced in relation to introducing this role into practice. One-to-one interviews provided an in-depth exploration of the phenomenon under study, while focus groups and interviews with more than one respondent enabled data to be generated through the social context of interaction between group members [40]. This allowed us to observe how group members responded to each other’s perspectives and elicited a frank discussion between respondents. This study took place in one metropolitan region within the UK and this may limit the transferability of findings to other (for example, more rural) settings. We were able to recruit SPLW stakeholder staff from five of six areas across the region where the role was operational, however our CN staff views come from only one area of five across the region where the role was operating and may be different from experiences in other Greater Manchester areas.

In addition, we only managed to recruit three GPs into the study sample. We highlight this as a limitation given that the reduction of GP workload is one of the key drivers for the introduction of these signposting roles. However, the small number of GPs that we did recruit provided valuable insights into how the roles were operating in practice.

We did not investigate how the CN or SPLW roles worked with other non-GP roles within general practice (such as practice nursing roles) as policy is focused on how such roles may or may not take away the work burden from GPs. This is a limitation of our study and future research could investigate how such roles function within the wider (non-GP) general practice team.

### Implications for research and practice

Insights from this study offer important learning in relation to process issues for future implementation of signposting via the roles of CN and SPLW that may be of benefit to those designing, planning or commissioning such services. The study emphasises the need for adequate communication and engagement between stakeholders to ensure clarity around role and service remit for both CN and SPLW roles and enable successful signposting. It also identifies a need for appropriate levels of training and skills development for role holders to enable roles to be carried out safely, effectively and ensure consistency in the offer across areas and sectors. The importance of partnership working and collaborative commissioning, including continued support for community and voluntary groups, in order to ensure capacity in the health and care system is maintained, to enable care remains person-centred is also highlighted. Importantly, our study brings to the fore the tension between the double aims in the CN and SPLW roles of signposting patients to the ‘right’ professional at the ‘right’ time, while simultaneously driving down the number of GP appointments by diverting patients away from GPs to other services. Policy-makers may need to consider how these tensions can be resolved, if the policy of ‘active signposting’ is to achieve its expressed aims. In addition, access to the ‘right’ professionals/services may not be available given the reports of pressurised services and the disconnect between parts of the system.

Data collection for this study took place just as the PCN SPLW roles were coming on stream. Future research could explore how PCN SPLW roles are being integrated into existing SPLW services and how the PCN employed SPLW service affects signposting/social prescribing in general practice.

## Conclusions

This study identifies barriers to integrating signposting into general practice and offers learning for those who may be planning such services. It highlights three key factors which can limit or enhance the operationalisation of signposting into primary care via the roles of CN and SPLW. Firstly, clarity about the purpose and remit of roles is needed. In particular we highlight the need to resolve the potential tension inherent the dual aims of ‘active signposting’ – to reduce GP workload while directing patients to the ‘right’ care. Secondly, we underline the importance of appropriate training and skills development for role holders to maximise the success of signposting in practice. Thirdly, adequate communication and engagement between stakeholders and partnership working across services are required to enable the integration of signposting into the general practice setting.

## Supplementary Information

**Additional file 1.** Interview topic guide (questions asked in semi-structured interviews/focus group).

## Data Availability

The datasets generated and/or analysed during this study are in the form of anonymised interview/focus group transcripts. Transcripts are not publically available but are held on a University of Manchester secure server in line with study ethical approval. Transcripts are available from the corresponding author on reasonable request.

## Abbreviations

ARRS: Additional Roles Reimbursement Scheme
CCG: Clinical Commissioning Group
CN: Care Navigator
CPD: Continuing Professional Development
DWP: Department of Work and Pensions
GP: General Practitioner
NHS LTP: National Health Service Long Term Plan
PCN: Primary Care Network
SPLW: Social Prescribing Link Worker
UK: United Kingdom
USA: United States of America
VCSE: Voluntary, Community and Social Enterprise
