# Supplementary material for: The challenges of integrating signposting into general practice: qualitative stakeholder perspectives on care navigation and social prescribing in primary care
Source: BMC Prim Care. 2022 Apr 1;23:66. doi: 10.1186/s12875-022-01669-z (PMC8972897; doi:10.1186/s12875-022-01669-z)
Supplement: Supplementary file 1 — Additional file 1. Interview topic guide (questions asked in semi-structured interviews/focus group). [file 12875_2022_1669_MOESM1_ESM.docx]

# Interview Schedule (WP3)

1. Describe how new roles have been introduced to general practice in your area.
2. What are the aims/objectives of introducing new roles?
3. What has been/is your role in facilitating this change?
4. What challenges have you encountered which have made it difficult to introduce new roles?
5. What is/was required to integrate new roles in general practice in your locality?
6. What steps have been taken so far?
7. How have you communicated the changes to staff/patients?
8. What challenges have been encountered (e.g. IT, IG, communications and engagement, workforce, finance, infrastructure)?
9. How have these challenges been tackled?
10. Are any early impacts emerging as a result of skill-mix changes in general practice?
11. What do you expect will be the impact of these changes (on patients, GPs, other staff and other parts of the health and social care system)?
12. How would you know if new roles have had the desired impact?
13. If the contribution of new roles is being evaluated, describe how that is being done.
14. How sustainable are the changes made in your area?
15. How is the wider context of new models of care impacting on the incorporation of new roles?
